# Patient-Reported Health-Related Quality of Life in Romanian Patients with Cystic Fibrosis in the Era of Highly Effective CFTR Modulator Therapy: A National Cross-Sectional Mixed-Methods Survey

**DOI:** 10.3390/jcm15166328

**Published:** 2026-08-16

**Authors:** Cristian Phillip Marinău, Cristian Sava, Alin Iuhas, Ioana Mihaiela Ciucă, Liviu Laurențiu Pop, Mihai Craiu, Zsolt Futaki, Ariana Szilagyi, Alexandru Jurca, Claudia Maria Jurca

**Affiliations:** 1 Department of Medical Disciplines, Faculty of Medicine and Pharmacy, University of Oradea, 410073 Oradea, Romania; 2 Pediatrics Department, Bihor County Clinical Emergency Hospital, 410167 Oradea, Romania; 3 Pediatric Department, University of Medicine and Pharmacy “Victor Babes”, 300041 Timișoara, Romania; 4 National Cystic Fibrosis Centre and Pediatric Pulmonology Ward, “Pius Brînzeu” County Emergency Clinical Hospital, 300226 Timișoara, Romania; 5 Department of Pediatrics, Carol Davila University of Medicine and Pharmacy, 050474 Bucharest, Romania; 6 National Institute for Mother and Child Health, Alessandrescu-Rusescu, 020395 Bucharest, Romania; 7 Department of Preclinical Disciplines, Faculty of Medicine and Pharmacy, University of Oradea, 410073 Oradea, Romania; 8 Regional Center of Medical Genetics Bihor, Bihor County Clinical Emergency Hospital (Part of ERN ITHACA), 410053 Oradea, Romania

**Keywords:** cystic fibrosis, health-related quality of life, CFTR modulators, patient-reported outcomes, mixed-methods survey

## Abstract

**Background/Objectives:** Highly effective cystic fibrosis transmembrane conductance regulator (CFTR) modulator therapy has changed cystic fibrosis (CF) care, but Romanian patient-reported health-related quality of life (HRQoL) data remain limited. This national cross-sectional mixed-methods survey aimed to describe patient- and parent-reported HRQoL in Romanian people with CF (pwCF) aged ≥6 years using age-appropriate Cystic Fibrosis Questionnaire-Revised (CFQ-R) versions, with the respiratory domain as the primary outcome. **Methods:** The online survey was conducted between February and April 2026. Respondents provided demographic and clinical data, including genotype/F508del status, CFTR modulator status, percent predicted forced expiratory volume in 1 s (ppFEV_1_) category, weight, height, and IV antibiotic-treated pulmonary exacerbations in the previous year. CFQ-R scores were summarized descriptively, and exploratory unadjusted associations were assessed using Mann–Whitney U tests and Spearman correlations. Open-ended responses were analyzed descriptively. **Results:** Of 67 responses, 61 were included: 43 participants aged 6–13 years and 18 aged ≥14 years. Most respondents were currently receiving CFTR modulators (52/61, 85.2%), predominantly elexacaftor/tezacaftor/ivacaftor. Mean CFQ-R Respiratory scores were 74.5 ± 23.1 in parent-proxy questionnaires for children aged 6–13 years, 83.3 ± 11.8 in self-reported questionnaires for children aged 12–13 years, and 69.8 ± 27.4 among respondents aged ≥14 years. Respiratory scores were higher among currently treated respondents and those with at least one F508del variant, and lower among those reporting IV antibiotic-treated exacerbations. Treatment burden remained among the lower-scoring domains. Qualitative responses described perceived respiratory, nutritional, and functional improvements, alongside residual treatment burden, psychosocial challenges, and access-related expectations. **Conclusions:** This national Romanian mixed-methods survey provides descriptive, exploratory CFQ-R-based HRQoL data in the CFTR modulator era. The findings suggest more favorable respiratory HRQoL among currently treated respondents, while supporting continued multidimensional patient-reported outcome monitoring in Romanian CF care.

## 1. Introduction

Cystic fibrosis (CF) is a life-limiting autosomal recessive disease caused by pathogenic variants in the cystic fibrosis transmembrane conductance regulator (*CFTR*) gene and is now estimated to affect approximately 188,000 people worldwide, although diagnosis and access to treatment remain highly unequal across countries [1,2]. Dysfunction of the CFTR protein impairs the transport of chloride and bicarbonate across the apical membrane of epithelial cells, contributing to airway surface dehydration, impaired mucociliary clearance, chronic suppurative lung disease, pancreatic insufficiency, and gastrointestinal and reproductive manifestations [2]. Even in the era of disease-modifying therapy, routine CF care commonly involves airway clearance, inhaled therapies, oral medications, pancreatic enzyme replacement, nutritional support, exercise, and recurrent monitoring, such that daily treatment workload remains substantial [3].

As survival improves and the demographic profile of CF broadens, health-related quality of life (HRQoL) has become an essential complement to conventional endpoints such as percent predicted forced expiratory volume in one second (ppFEV_1_), body mass index (BMI), or pulmonary exacerbation history [2]. Among disease-specific instruments, the Cystic Fibrosis Questionnaire-Revised (CFQ-R) is the most widely used tool across pediatric, adolescent, adult, and parent-proxy populations, assessing respiratory symptoms alongside broader domains such as physical functioning, emotional well-being, eating-related difficulties, body image, social participation, and treatment burden [4]. The instrument provides multidomain scores ranging from 0 to 100, with higher scores indicating better HRQoL, and its respiratory domain has particular interpretive value because a minimal clinically important difference has been defined for clinically meaningful change [4,5]. Electronic versions of the CFQ-R have also been validated and are available in multiple languages [6,7].

The modulator era has substantially changed the context in which CFQ-R findings should be interpreted. In pivotal phase 3 trials, elexacaftor/tezacaftor/ivacaftor (ETI) was associated with marked improvements in the CFQ-R Respiratory domain among adolescents and adults with either one F508del allele plus a minimal-function variant or homozygous F508del disease [8,9]. Pediatric studies extended this signal to younger patients, including children aged 6–11 years, and longer-term extension data support sustained benefit in F508del-eligible groups [10,11].

At the same time, improved respiratory status does not imply complete normalization of lived experience. Modulator-era studies suggest that treatment burden may remain one of the more vulnerable HRQoL domains, even when respiratory and physical domains improve, and that patients continue to weigh uncertainty around long-term simplification of legacy daily regimens [2,12,13]. Broader issues such as emotional functioning, mental health, and well-being also remain clinically relevant in the modulator era [14,15]. In parallel, the treatment landscape is still shaped by genotype eligibility, reimbursement policy, and unequal access. Within Europe, the therapeutic indication for ETI has progressively expanded, and Romania has likewise broadened reimbursement through successive therapeutic protocol updates since 2022 [16,17,18,19,20]. This context matters for HRQoL interpretation, because patients who are not currently receiving CFTR modulators may differ not only in respiratory burden, but also in perceived health, expectations, and uncertainty regarding future treatment access [1,16].

The Eastern European evidence base remains smaller than that from North America and Western Europe. Pre-modulator studies from Hungary and Poland showed that CFQ-R-assessed HRQoL was shaped by disease severity, lung function, nutritional status, school participation, and differences between child- and parent-reported perceptions [21,22]. More recent Polish work has added contemporary cross-sectional CFQ-R data and pediatric post-approval ETI outcomes, suggesting that regional evidence is growing but remains relatively fragmented [23,24]. In Romania, published CF research has addressed psychosocial burden in children, determinants of lung function, and related clinical dimensions of disease, while a recent adult Romanian study has examined quality of life (QoL) alongside mental health and sleep [14,25,26]. Nevertheless, national data using age-specific CFQ-R versions to characterize HRQoL in Romanian people with Cystic Fibrosis (pwCF) during the modulator era remain sparse.

To address this gap, the present national cross-sectional mixed-methods survey aimed to describe patient- and parent-reported HRQoL in Romanian pwCF aged ≥6 years using age-appropriate CFQ-R versions. The CFQ-R Respiratory domain served as the primary outcome, while other domains and clinical variables—including genotype, ppFEV_1_, BMI, exacerbations, and CFTR modulator status—were examined exploratorily. Additionally, open-ended responses contextualized the quantitative data by capturing patient-centered perspectives on treatment changes, residual challenges, expectations, and access-related concerns.

## 2. Materials and Methods

### 2.1. Study Design and Population

The study was designed as a national, cross-sectional, observational mixed-methods online survey of pwCF from Romania. Eligible participants were patients with a diagnosis of cystic fibrosis aged 6 years or older, pediatric and adult patients alike. Responses from participants younger than 6 years of age or with incomplete key data required for analysis were excluded.

The questionnaire was created using Google Forms (Google LLC, Mountain View, CA, USA), and the survey link was distributed through open and overlapping channels, including social media, a cystic fibrosis patient association, and cystic fibrosis centers; participants were instructed to submit only one response each. Recruitment was non-probability and volunteer-based and may therefore have preferentially reached families who were digitally connected, engaged with patient organizations, or in contact with participating centers, thereby limiting the representativeness of the sample. Because the dissemination strategy did not permit identification of the number of unique individuals who viewed or received the invitation, the recruitment denominator and response rate could not be calculated. The online questionnaire was available from February to April 2026.

### 2.2. Data Collection and Variables

The survey collected self-reported demographic and clinical data, which were not independently verified against medical records, regarding the following:

- Demographic Characteristics: Age, sex, residence (urban/rural).
- Nutritional Status: Weight and height. The BMI was calculated based on the self-reported weight and height, and z-scores were based on reference values from Centers for Disease Control and Prevention.
- Genetic Profile: Homozygous F508del, heterozygous F508del, no F508del, and unknown.
- CFTR Modulator Therapy Status: Currently treated, interrupted and never treated. CFTR modulator therapy duration in months.
- Lung Function: ppFEV_1_ as >70%; 40–69%; <40%; unknown.
- Pulmonary Exacerbation Frequency: Number of exacerbations that required iv antibiotic treatment in the last 12 months.
- QoL: Using age-specific CFQ-R.
- Open-Ended Questions: Perceived treatment-related changes, residual challenges, and future expectations.

Each participant contributed one age-appropriate CFQ-R questionnaire. For children aged 6–13 years, responses were provided primarily by caregivers using the parent-proxy version; a small subgroup of children aged 12–13 years completed the self-report version. Respondents aged ≥14 years completed the teen/adult self-report version.

Domain scores of the CFQ-R were calculated using the online scoring software developed by the University Hospital of Copenhagen [7].

### 2.3. Analysis

Statistical analysis was performed using Microsoft Excel 2021 (Microsoft Corporation, Redmond, WA, USA) and JASP (Version 0.96.0; JASP Team, Amsterdam, the Netherlands).

Patient characteristics and CFQ-R domain scores were summarized descriptively as n/N (%) for categorical variables and mean ± standard deviation (SD) for continuous variables; CFQ-R Respiratory scores were additionally reported as median and interquartile range (IQR), where appropriate. Group comparisons were performed using the Mann–Whitney U test, and associations with BMI, BMI z-score, and duration of current CFTR modulator therapy were assessed using Spearman’s rank correlation coefficient.

The CFQ-R Respiratory domain was the primary HRQoL outcome, whereas non-respiratory domain analyses were exploratory. Participants with interrupted or no previous CFTR modulator therapy were combined into the not currently treated group. Because some subgroups contained fewer than 10 participants, there comparisons were interpreted cautiously and were not considered confirmatory. Analyses were based on available cases without imputation. Exact denominators are reported for each analysis; missing data affected age (*n* = 1), sex (*n* = 2), residence (*n* = 1), exacerbation history (*n* = 2), and BMI (*n* = 2), while pediatric BMI z-score was available for 40/43 children. In addition, 24 participants reported unknown ppFEV_1_ category and 3 reported unknown F508del status; these were retained descriptively but excluded from the corresponding inferential analyses. All inferential analyses were exploratory and unadjusted. No correction for multiple comparisons was applied; therefore, nominal *p*-values should be interpreted cautiously because of the increased probability of chance findings, with *p* < 0.05 considered statistically significant.

Open-ended responses were analyzed using content analysis [27], combining a deductive framework based on the study objectives with inductive identification of additional themes. Two researchers (C.P.M. and A.I.) independently categorized responses and resolved disagreements through discussion. Responses could contribute to more than one theme, and theme frequencies were calculated using participants with available qualitative responses as the denominator. Qualitative findings were interpreted descriptively as contextual and hypothesis-generating.

### 2.4. Disclosure of Generative AI Use

Generative artificial intelligence (ChatGPT Model 5.5) was utilized during the preparation of this manuscript for the purpose of technical text editing, structural organization, and translation assistance to ensure academic linguistic standards.

## 3. Results

### 3.1. Respondent Characteristics

Of 67 submitted responses, 6 were excluded (3 from participants aged <6 years and 3 because of incomplete key data), leaving 61 respondents for analysis: 43 aged 6–13 years and 18 aged ≥14 years (Table 1). Exact age was available for 60 respondents, with a mean age of 13.0 ± 7.8 years.

Most respondents reported at least one F508del variant and were currently receiving CFTR modulator therapy, predominantly ETI, with 2 patients receiving lumacaftor/ivacaftor. Among currently treated respondents, the median duration of therapy was 28.5 months (IQR 14.0–36.0). Self-reported ppFEV_1_ was unknown for a substantial proportion of participants, whereas approximately one-third of respondents with available data reported at least one IV antibiotic-treated pulmonary exacerbation during the preceding 12 months. Detailed demographic, clinical, lung-function, and nutritional characteristics are presented in Table 1.

### 3.2. CFQ-R Domain Scores

**Figure 1:**
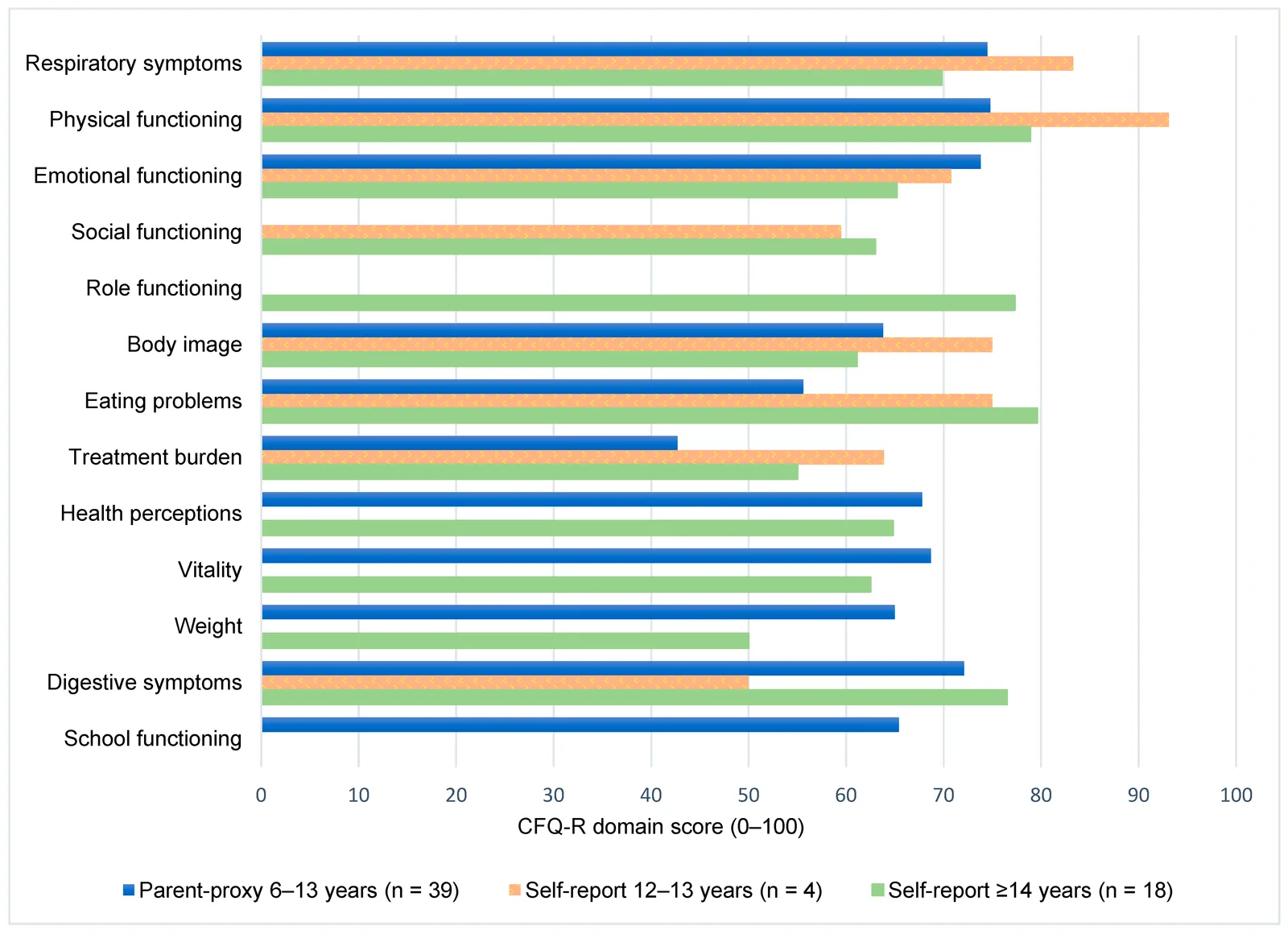
Mean CFQ-R domain scores by questionnaire version. CFQ-R, Cystic Fibrosis Questionnaire-Revised. CFQ-R domain scores range from 0 to 100, with higher scores indicating better health-related quality of life. Domains are shown only when included in the corresponding age-specific CFQ-R version. Comparisons across questionnaire versions should be interpreted descriptively because respondent type and available domains differ. In particular, estimates for the 12–13-year self-report subgroup (*n* = 4) should be interpreted with caution.

CFQ-R domain scores by questionnaire version are summarized in Figure 1 and Table 2. Scores range from 0 to 100, with higher scores indicating better HRQoL.

For the primary outcome, mean CFQ-R Respiratory domain scores were 74.5 ± 23.1 in parent-reported questionnaires for children aged 6–13 years (*n* = 39), 83.3 ± 11.8 in self-reported questionnaires for children aged 12–13 years (*n* = 4), and 69.8 ± 27.4 in self-reported questionnaires for respondents aged ≥14 years (*n* = 18).

Across questionnaire versions, treatment burden was among the lower-scoring domains, whereas physical functioning, respiratory symptoms, and several other domains generally showed moderate-to-high mean scores (Figure 1, Table 2).

### 3.3. Exploratory Analysis of CFQ-R Respiratory Domain

Exploratory subgroup analyses of the CFQ-R Respiratory domain are summarized in Table 3. All analyses were unadjusted and based on available cases.

Respiratory domain scores were higher among respondents currently receiving CFTR modulator therapy than among those not currently treated (*p* = 0.007) and among participants with at least one F508del variant compared with those without F508del (*p* = 0.014). Both comparison groups included small subgroups—9 respondents were not currently treated and 8 had no F508del variant—and these findings should therefore be interpreted cautiously. No clear differences in respiratory scores were observed according to age group, sex, or residence (Table 3).

Among respondents with known ppFEV_1_, respiratory scores were numerically higher in those reporting ppFEV_1_ > 70% predicted than in those reporting ppFEV_1_ 40–69% predicted, but the difference was not statistically clear (*p* = 0.328). This comparison was particularly limited by the small number of respondents in the 40–69% category (*n* = 6) and by the high proportion of participants with unknown ppFEV_1_.

Participants reporting at least one IV antibiotic-treated pulmonary exacerbation in the previous 12 months had lower respiratory scores than those reporting no such exacerbation (*p* = 0.046). BMI z-score was not clearly associated with respiratory score among children aged 6–13 years, whereas a positive correlation between BMI and respiratory score was observed among respondents aged ≥14 years (ρ = 0.65, *p* = 0.005). Duration of current CFTR modulator therapy was not clearly associated with respiratory score (Table 3).

### 3.4. Qualitative Findings

Open-ended responses with analyzable content were available for 49/52 respondents currently receiving CFTR modulator therapy and 3/9 respondents not currently receiving modulator therapy (Supplementary Table S1). Codes were non-mutually exclusive and were analyzed descriptively.

Among respondents currently receiving CFTR modulators, the dominant pattern was perceived clinical improvement after treatment initiation, especially respiratory improvement. Respondents most often reported less cough or easier breathing (16/49, 32.7%), fewer respiratory infections or milder viral episodes (14/49, 28.6%), and fewer hospitalizations or antibiotic courses (14/49, 28.6%). Other frequently described benefits included clear overall improvement (13/49, 26.5%), weight gain or better growth (13/49, 26.5%), and more energy or less fatigue (12/49, 24.5%). Some respondents also reported improved daily activities, school participation, laboratory or sweat-test results, and digestive symptoms.

However, benefits were not universal. A minority reported no major change (5/49, 10.2%) or continuing challenges, including daily treatment burden, anxiety or stress, treatment fatigue, family burden, stigma, side effects, or other comorbidities. Among respondents not currently receiving CFTR modulator therapy, responses focused on access and future expectations, including waiting for modulator eligibility (2/3, 66.7%) and desire for new medicines or better access (1/3, 33.3%).

### 3.5. Exploratory Analysis of Non-Respiratory CFQ-R Domains

Exploratory associations across non-respiratory CFQ-R domains are summarized in Supplementary Table S2. All analyses were unadjusted and based on available cases; given the number of comparisons performed and the absence of correction for multiple testing, the findings should be interpreted as hypothesis-generating.

Among children aged 6–13 years, BMI z-score was not clearly associated with non-respiratory CFQ-R domains. Among respondents aged ≥14 years, higher BMI showed positive correlations with several domains, including emotional functioning, role functioning, body image, and weight. These analyses were based on 17 respondents and should therefore be interpreted cautiously.

Exploratory comparisons according to CFTR modulator status, F508del status, ppFEV_1_ category, and IV antibiotic-treated pulmonary exacerbations suggested differences across selected physical, emotional, functional, and health-perception domains (Supplementary Table S2). In particular, respondents reporting a recent IV antibiotic-treated exacerbation tended to have less favorable scores across several domains. Several of these analyses involved small subgroups, including only six respondents in the ppFEV_1_ 40–69% category, and no adjustment was made for multiple comparisons; individual nominal *p*-values should therefore not be interpreted as confirmatory evidence.

## 4. Discussion

To our knowledge, this is the first national mixed-methods study to describe HRQoL in both children and adults with cystic fibrosis from Romania in the era of CFTR modulator therapy. This is relevant because, in general, real-world data regarding HRQoL in Romanian pwCF remain limited [14,25].

Most of our respondents were caregivers of children aged 6–13 years and the majority were currently receiving CFTR modulator therapy, predominantly ETI. Overall, CFQ-R Respiratory domain scores suggested moderate-to-high perceived respiratory QoL, although scores varied across age-specific questionnaire versions and respondent groups. Treatment burden was among the lowest-scoring domains, indicating that daily therapeutic demands persist despite widespread modulator use. In exploratory unadjusted analyses, CFQ-R Respiratory scores were higher among respondents currently receiving CFTR modulators and among those with at least one F508del variant, although both comparisons involved small comparator groups. Lower scores were observed among participants reporting at least one IV antibiotic-treated pulmonary exacerbation in the previous 12 months. No clear differences in respiratory scores were observed by age group, sex, residence, or ppFEV_1_ category, and duration of current modulator therapy was not clearly associated with respiratory quality of life.

Among respondents aged ≥14 years, higher BMI correlated with better respiratory and several non-respiratory CFQ-R domains, while BMI z-score in children was not clearly associated with CFQ-R scores. Qualitative responses largely supported the quantitative findings, with treated respondents commonly reporting perceived improvements in cough, breathing, infections, hospitalizations, weight or growth, energy, and daily functioning, while also highlighting persistent treatment burden, emotional stress, treatment fatigue, side effects, and access-related expectations.

### 4.1. Respiratory Domain and Modulator-Era Comparison

The Respiratory-domain findings of the present study are consistent with the broader modulator-era literature showing that CFTR modulator therapy is associated with better patient-reported respiratory health. In this Romanian cohort, CFQ-R Respiratory domain scores were higher among respondents currently receiving CFTR modulator therapy than among those not currently treated, and qualitative responses frequently described less cough, easier breathing, fewer respiratory infections, and reduced need for hospitalization or antibiotics. This pattern aligns with the PROMISE study, which evaluated ETI in a real-world setting and included the CFQ-R Respiratory domain among its clinical effectiveness outcomes [28]. Similar improvements in patient-reported respiratory or HRQoL outcomes have been reported in adolescent and adult real-world cohorts after ETI initiation, including an Irish prospective real-world study incorporating CFQ-R and other patient-reported outcomes, and a Dutch prospective observational study using CFQ-R and novel QoL instruments [29,30]. Taken together, these studies support the interpretation that the higher respiratory scores observed among currently treated respondents in our survey are directionally compatible with modulator-era real-world evidence, although the cross-sectional design of the present study precludes causal inference.

Treatment status was closely related to genotype eligibility and access to therapy, as most treated respondents carried at least one F508del variant, and participants with at least one F508del variant also had higher CFQ-R Respiratory scores than those without F508del. Pediatric and genotype-specific studies from the modulator era further support the relevance of CFQ-R Respiratory outcomes in younger patients and F508del-eligible groups, including phase 3 open-label ETI studies in children aged ≥6 years [10,11], and pediatric observational studies evaluating QoL after ETI initiation [31,32]. At the same time, the lower respiratory scores observed among respondents reporting IV antibiotic-treated pulmonary exacerbations in the previous 12 months are clinically plausible and consistent with earlier CFQ-R literature linking respiratory symptom burden, healthcare utilization, and poorer HRQoL [5,33].

Although clinically relevant, the not currently treated subgroup was small (*n* = 9), and comparisons involving this group should therefore be regarded as exploratory. These respondents had lower CFQ-R Respiratory scores than those currently receiving CFTR modulators, and exploratory analyses also suggested less favorable selected non-respiratory scores, particularly vitality, Health perceptions, and Role functioning. The limited qualitative responses available from participants not currently receiving modulator therapy focused mainly on eligibility and hopes for improved treatment access. These findings suggest that, in the modulator era, HRQoL among patients not currently treated may be shaped by respiratory burden, daily functioning, perceived health, uncertainty, and access-related expectations. This is consistent with studies showing that ETI may influence both HRQoL and daily treatment decisions, as well as broader well-being in people with CF and caregivers [12,34].

### 4.2. Non-Respiratory Domains and Qualitative Findings

Our data indicate that treatment burden was among the lowest-scoring domains, particularly in parent-reported questionnaires for children aged 6–13 years and in respondents aged ≥14 years, whereas physical functioning and several symptom-related domains showed comparatively higher scores. This pattern is consistent with modulator-era literature indicating that highly effective CFTR modulator therapy may improve several patient-reported outcomes, but does not necessarily eliminate the everyday burden of treatment, monitoring, symptoms, and disease-related restrictions [3,13,32]. In this context, respiratory and physical domains appeared generally more favorable, whereas treatment burden remained low and vitality showed only moderate scores, suggesting that respiratory improvement may not fully translate into broader perceived well-being.

This uneven HRQoL profile may also be interpreted alongside ETI-era biomarker studies. In the PROMISE-Inflammation substudy, Sagel et al. reported sustained reductions in airway inflammatory markers and circulating high sensitivity C-reactive protein during long-term ETI therapy, with changes in inflammatory markers correlating with improvements in ppFEV_1_ and respiratory symptoms [35]. However, respiratory improvement may not fully reflect systemic recovery, as Örlős et al. found that systemic redox status remained largely unchanged after six months of ETI, despite reduced airway oxidative stress [36]. These findings may help contextualize the persistence of lower vitality, treatment burden, or other non-respiratory concerns despite relatively favorable respiratory scores.

Psychological factors may also contribute to incomplete recovery in selected HRQoL domains. In the present study, emotional functioning and social functioning showed only moderate scores in some respondent groups, and IV antibiotic-treated exacerbations were associated with lower emotional functioning and vitality. Qualitative responses further described anxiety or stress, treatment fatigue, family burden, and peer-related stigma. Recent discussions have addressed depression-related symptoms reported during ETI therapy, but available evidence does not support a clear causal relationship between ETI and depression-related events [37,38]. Therefore, lower vitality or incomplete improvement in perceived well-being should be interpreted as potentially multifactorial, reflecting chronic disease burden, treatment demands, exacerbation history, psychosocial factors, and comorbidities, rather than being attributed directly to modulator therapy.

The observed positive correlations between BMI and several domains among respondents aged ≥14 years, including emotional functioning, role functioning, body image, weight, and respiratory symptoms, are also clinically plausible and align with previous literature linking nutritional status, body composition, and perceived functioning with QoL in CF [39,40].

The qualitative findings provided complementary context to the CFQ-R results. Among respondents currently receiving CFTR modulators, perceived benefits extended beyond respiratory improvement and included better weight or growth, more energy, improved daily activity or school participation, digestive improvement, and a general sense of better health. These themes are consistent with mixed-methods and real-world modulator-era studies showing that ETI may reshape patients’ perceptions of daily functioning, wellbeing, and treatment routines [12,34]. At the same time, the persistence of treatment burden, anxiety or stress, treatment fatigue, family burden, stigma, side effects, and access-related expectations in some responses highlights that improved clinical or respiratory status does not necessarily translate into complete normalization of lived experience. Similar concerns have been emphasized in studies addressing treatment burden and mental health in the modulator era, where patients may experience substantial benefit while continuing to face psychosocial, and adherence-related challenges [12,13,15]. Therefore, the qualitative data should be interpreted as patient- and caregiver-reported perceptions rather than evidence of treatment effectiveness, but they strengthen the interpretation that CF care in Romania should continue to assess multidimensional QoL alongside respiratory outcomes.

### 4.3. Regional and Local Context

Prior to the widespread availability of highly effective CFTR modulators, data regarding HRQoL in CF within Eastern Europe were limited and primarily derived from observational studies. Pre-modulator studies from Poland and Hungary showed that HRQoL in children, adolescents, and adults with CF was associated with disease severity, respiratory and nutritional status, psychosocial functioning, and differences between patient- and parent-reported perceptions [21,22,41]. Contemporary HRQoL evidence from the region is gradually emerging. A recent Polish cross-sectional study described CFQ-R-assessed HRQoL and its influencing factors in patients aged 6–42 years, while a Polish post-approval pediatric ETI study evaluated QoL outcomes together with pulmonary and nutritional parameters [23,24].

A similar scarcity of pre-modulator psychosocial data exists in Romania. Early work by Anton-Paduraru et al. highlighted the emotional, social, and family-related dimensions of the disease burden among children at a regional center in northeastern Romania [25]. Other Romanian clinical studies have emphasized the broader burden of CF through organ-specific manifestations and clinical determinants, including sinonasal complications and factors associated with impaired lung function [26,42]. While the existing Romanian CF literature encompasses psychosocial assessments, mental health studies, and case reports on complex clinical scenarios [14,25,43,44], national CFQ-R-based HRQoL data in the CFTR modulator era remain notably sparse. Recent Romanian work has begun to address mental health, sleep quality, and QoL in adults with CF, including a randomized controlled trial evaluating progressive muscle relaxation as part of pulmonary rehabilitation [14]. Therefore, the present national Romanian mixed-methods survey contributes regionally relevant patient-reported evidence by describing respiratory and non-respiratory CFQ-R domains, together with perceived benefits, residual treatment burden, psychosocial challenges, and access-related expectations during the evolving CFTR modulator era.

### 4.4. Strengths and Limitations

The main strengths of this study are its national scope, use of age-specific CFQ-R versions, and mixed-methods design, which allowed quantitative HRQoL scores to be contextualized through patient- and caregiver-reported experiences. Nevertheless, the findings should be interpreted cautiously. The cross-sectional design precludes causal inference, recruitment methods may have introduced selection bias, and the modest sample size limited subgroup analyses. In addition, clinical variables were self-reported, ppFEV_1_ was frequently unknown, analyses were available-case and unadjusted, and the use of both proxy and self-report CFQ-R versions limits direct comparability across age groups. Therefore, the results should be considered descriptive and hypothesis-generating.

### 4.5. Future Directions

Future research should build on these exploratory findings through longitudinal, multicenter Romanian studies integrating repeated CFQ-R assessments with objective clinical measures, including ppFEV_1_, nutritional status, exacerbation history, genotype, treatment type, and treatment duration. Such studies could clarify the durability of respiratory HRQoL benefits among patients receiving CFTR modulators and support routine HRQoL monitoring in CF care. Further research should also address persistent treatment burden, including treatment time, volume, complexity, cost, and logistical challenges, from both caregiver and adult patient perspectives. In addition, dedicated studies are needed for patients who are not eligible for CFTR modulator therapy and for children younger than 6 years, as these groups may have distinct HRQoL needs and remain underrepresented in current Romanian data.

## 5. Conclusions

This national Romanian mixed-methods survey provides descriptive evidence on CFQ-R-assessed HRQoL in the CFTR modulator era. Respondents receiving CFTR modulators reported more favorable respiratory QoL and perceived broader benefits, while the small not currently treated group had lower respiratory scores and expressed access- and eligibility-related expectations. Treatment burden and psychosocial challenges remained relevant across the cohort. Given its cross-sectional and exploratory design, this study should be interpreted cautiously and may serve as a foundation for future longitudinal Romanian HRQoL research and routine patient-reported outcome monitoring in CF care.

## Figures and Tables

**Table 1:** Demographic and Clinical Characteristics of the Study Population.

Characteristic	Overall	Age 6–13 Years	Age ≥ 14 Years
Participants, N	61	43	18
Age (years), mean ± SD	13.0 ± 7.8, (*n* = 60)	8.9 ± 2.5 (*n* = 42)	22.8 ± 7.5 (*n* = 18)
Sex, n/N (%)			
Female	33/59 (56.0%)	25/42 (59.5%)	8/17 (47.0%)
Male	26/59 (44.0%)	17/42 (40.5%)	9/17 (53.0%)
Residence, n/N (%)			
Urban	44/60 (73.3%)	31/42 (73.8%)	13/18 (72.2%)
Rural	16/60 (26.7%)	11/42 (26.2%)	5/18 (27.8%)
F508del status, n/N (%)			
Homozygous F508del	22/61 (36.1%)	12/43 (27.9%)	10/18 (55.6%)
Heterozygous F508del	28/61 (45.9%)	23/43 (53.5%)	5/18 (27.8%)
No F508del	8/61 (13.1%)	7/43 (16.3%)	1/18 (5.6%)
Unknown	3/61 (4.9%)	1/43 (2.3%)	2/18 (11.1%)
CFTR modulator therapy status n/N (%)			
Currently treated	52/61 (85.2%)	36/43(83.7%)	16/18 (88.9%)
Interrupted	1/61 (1.6%)	1/43 (2.3%)	0/18 (0.00%)
Never treated	8/61 (13.1%)	6/43 (14.0%)	2/18 (11.1%)
Duration of current CFTR modulator therapy, months, median (IQR)	28.5 (14.0–36.0), *n* = 52	24 (13.8–35.3), *n* = 36	36 (22.5–46), *n* = 16
ppFEV_1_, n/N (%)			
>70%	31/61 (50.8%)	21/43 (48.8%)	10/18 (55.6%)
40–69%	6/61 (9.8%)	3/43 (7.0%)	3/18 (16.7%)
<40%	0/61 (0.00%)	0/43 (0.00%)	0/18 (0.00%)
Unknown	24/61 (39.3%)	19/43 (44.2%)	5/18 (27.8%)
IV antibiotic-treated pulmonary exacerbation (past 12 month), n/N (%)			
No	39/59 (66.1%)	26/41 (63.4%)	13/18 (72.2%)
Yes	20/59 (33.9%)	15/41 (36.6%)	5/18 (27.8%)
Nutritional status			
BMI, kg/m^2^, mean ± SD	17.7 ± 3.3, *n* = 59	16.6 ± 2.9, *n* = 42	20.5 ± 2.7, *n* = 17
BMI z-score, mean ± SD		−0.2 ± 1.6, *n* = 40	

Abbreviations: BMI, body mass index; CFTR, cystic fibrosis transmembrane conductance regulator; IQR, interquartile range; ppFEV_1_, percent predicted forced expiratory volume in 1 s; SD, standard deviation. Data are presented as n/N (%) for categorical variables, mean ± SD for continuous variables, and median (IQR) for duration of current CFTR modulator therapy. In n/N (%), n represents the number of participants in the category and N represents the number of participants with available data for the corresponding variable. Percentages were calculated using available-case denominators. Unknown responses, including unknown ppFEV_1_ category, indicate data that were not known or not recalled by respondents at the time of survey completion and were retained as separate descriptive categories where applicable. BMI z-score was reported only for participants aged 6–13 years.

**Table 2:** CFQ-R domain scores by questionnaire version and age group.

CFQ-R Domain	Parent-Reported, Age 6–13 Years, Mean ± SD	Self-Reported, Age 12–13 Years, Mean ± SD	Self-Reported, ≥14 Years, Adolescents and Adults, Mean ± SD
Participants, N	39	4	18
Primary outcome			
Respiratory symptoms	74.5 ± 23.1	83.3 ± 11.8	69.8 ± 27.4
Other CFQ-R domains			
Physical functioning	74.8 ± 20.1	93.1 ± 10.5	78.9 ± 28.5
Emotional functioning	73.8 ± 22.7	70.8 ± 23.3	65.2 ± 23.5
Social functioning	—	59.5 ± 13.7	63.0 ± 25.6
Role functioning	—	—	77.3 ± 24.4
Body image	63.8 ± 30.0	75.0 ± 50.0	61.1 ± 33.3
Eating problems	55.6 ± 30.2	75.0 ± 22.9	79.6 ± 22.6
Treatment burden	42.7 ± 23.4	63.9 ± 31.9	55.0 ± 20.3
Health perceptions	67.8 ± 22.6	—	64.8 ± 20.6
Vitality	68.7 ± 17.0	—	62.5 ± 21.6
Weight	65.0 ± 39.0	—	50.0 ± 38.3
Digestive symptoms	72.1 ± 21.3	50.0 ± 43.0	76.5 ± 19.4
School functioning	65.4 ± 23.8	—	—

Abbreviations: CFQ-R, Cystic Fibrosis Questionnaire-Revised. CFQ-R domain scores are presented as mean ± SD and range from 0 to 100, with higher scores indicating better health-related quality of life. Em dashes (—) indicate domains that are not included in the corresponding age-specific CFQ-R questionnaire version and were therefore not assessed; they do not represent missing data.

**Table 3:** Exploratory associations with CFQ-R Respiratory domain.

Variable	Category/Analysis Group	N	CFQ-R RD Score, Mean ± SD	Median (IQR)	Exploratory *p*-Value
Age group	6–13 years	43	75.3 ± 22.3	83.3 (63.9–91.7)	*p* 0.520
	≥14 yeas	18	69.7 ± 27.4	77.8 (57.0–88.9)	
Sex	Female	33	70.8 ± 26.8	77.8 (44.4–91.7)	*p* 0.684
	Male	26	76.4 ± 20.2	80.6 (62.5–91.0)	
Residence	Urban	44	70.3 ± 25.9	77.8 (52.8–88.9)	*p* 0.208
	Rural	16	81.8 ± 15.0	86.1 (70.8–94.4)	
CFTR modulator therapy status	Currently treated	52	77.5 ± 20.9	83.3 (65.3–94.4)	
	Not currently treated *	9	51.8 ± 29.0	55.6 (27.8–77.8)	*p* 0.007
F508del status	At least one F508del ** variant	50	78.2 ± 20.2	83.3 (66.7–94.4)	*p* 0.014
	No F508del	8	54.2 ± 29.1	63.9 (31.9–73.6)	
ppFEV_1_ category	>70% predicted	31	80.6 ± 19.5	88.9 (75.0–94.4)	*p* 0.328
	40–69% predicted	6	62.0 ± 37.9	66.7 (38.9–94.4)	
IV antibiotic-treated pulmonary exacerbation in previous 12 months	No	39	77.7 ± 21.1	83.3 (66.7–94.4)	*p* 0.046
	Yes	20	63.9 ± 27.0	75.0 (41.7–83.3)	
BMI z-score, age 6–13 years	Spearman correlation	40	—	—	*ρ* ^†^ 0.02, *p* 0.902
BMI, age ≥ 14 years	Spearman correlation	17	—	—	*ρ* 0.65, *p* 0.005
Duration of current CFTR modulator therapy, months	Spearman correlation among currently treated participants	52	—	—	*ρ* 0.17, *p* 0.221

For the age-group analysis, the 6–13 years category (N = 43) combines CFQ-R respiratory domain scores from the parent-proxy questionnaire completed for children aged 6–13 years (*n* = 39) and the self-report questionnaire completed by children aged 12–13 years (*n* = 4). * The “not currently treated” CFTR modulator group included one participant who had interrupted treatment at the time of questionnaire completion. ** For F508del status, homozygous and heterozygous F508del participants were grouped as having at least one F508del variant; participants with unknown F508del status were excluded from this comparison. ^†^ Spearman’s rho.

## Data Availability

The original data presented in the study are openly available at https://doi.org/10.5281/zenodo.21103316.

## Supplementary Materials

The following supporting information can be downloaded at: https://www.mdpi.com/article/10.3390/jcm15166328/s1, Table S1. Categories, codes, frequencies, and illustrative quotations from qualitative content analysis of open-ended responses by CFTR modulator therapy status; Table S2. Exploratory associations across non-respiratory CFQ-R domains.

## Abbreviations

The following abbreviations are used in this manuscript:

BMI	body mass index
CF	cystic fibrosis
CFQ-R	cystic fibrosis questionnaire-revised
CFTR	cystic fibrosis transmembrane conductance regulator
ETI	elexacaftor/tezacaftor/ivacaftor
HRQoL	health-related quality of life
IQR	interquartile range
ppFEV_1_	percent predicted forced expiratory volume in 1 s
pwCF	people with cystic fibrosis
SD	standard deviation
QoL	quality of life

## References

[1] Guo J., King I., Hill A. (2024). International Disparities in Diagnosis and Treatment Access for Cystic Fibrosis. Pediatr. Pulmonol..

[2] Mall M.A., Burgel P.-R., Castellani C., Davies J.C., Salathe M., Taylor-Cousar J.L. (2024). Cystic Fibrosis. Nat. Rev. Dis. Primer.

[3] Altabee R., Mwamba M.J., Turner D., Davies G., Abbott J., Simmonds N.J., Whitty J.A., Carr S.B., Barton G., Cameron R.A. (2025). Measurement of Treatment Burden in Cystic Fibrosis: A Systematic Review. J. Cyst. Fibros..

[4] Quittner A.L., Buu A., Messer M.A., Modi A.C., Watrous M. (2005). Development and Validation of the Cystic Fibrosis Questionnaire in the United States: A Health-Related Quality-of-Life Measure for Cystic Fibrosis. Chest.

[5] Quittner A.L., Modi A.C., Wainwright C., Otto K., Kirihara J., Montgomery A.B. (2009). Determination of the Minimal Clinically Important Difference Scores for the Cystic Fibrosis Questionnaire-Revised Respiratory Symptom Scale in Two Populations of Patients with Cystic Fibrosis and Chronic Pseudomonas Aeruginosa Airway Infection. Chest.

[6] Solé A., Olveira C., Pérez I., Hervás D., Valentine V., Yepez A.N.B., Olveira G., Quittner A.L. (2018). Development and Electronic Validation of the Revised Cystic Fibrosis Questionnaire (CFQ-R Teen/Adult): New Tool for Monitoring Psychosocial Health in CF. J. Cyst. Fibros..

[7] Ronit A., Gelpi M., Argentiero J., Mathiesen I., Nielsen S.D., Pressler T., Quittner A.L. (2017). Electronic Applications for the CFQ-R Scoring. Respir. Res..

[8] Middleton P.G., Mall M.A., Dřevínek P., Lands L.C., McKone E.F., Polineni D., Ramsey B.W., Taylor-Cousar J.L., Tullis E., Vermeulen F. (2019). Elexacaftor-Tezacaftor-Ivacaftor for Cystic Fibrosis with a Single Phe508del Allele. N. Engl. J. Med..

[9] Heijerman H.G.M., McKone E.F., Downey D.G., Van Braeckel E., Rowe S.M., Tullis E., Mall M.A., Welter J.J., Ramsey B.W., McKee C.M. (2019). Efficacy and Safety of the Elexacaftor/Tezacaftor/Ivacaftor Combination Regimen in People with Cystic Fibrosis Homozygous for the F508del Mutation: A Double-Blind, Randomised, Phase 3 Trial. Lancet Lond. Engl..

[10] Zemanick E.T., Taylor-Cousar J.L., Davies J., Gibson R.L., Mall M.A., McKone E.F., McNally P., Ramsey B.W., Rayment J.H., Rowe S.M. (2021). A Phase 3 Open-Label Study of Elexacaftor/Tezacaftor/Ivacaftor in Children 6 through 11 Years of Age with Cystic Fibrosis and at Least One F508del Allele. Am. J. Respir. Crit. Care Med..

[11] Mall M.A., Wainwright C.E., Legg J., Chilvers M., Gartner S., Dittrich A.-M., Stehling F., Conner S., Grant S., Suresh N. (2025). Elexacaftor/Tezacaftor/Ivacaftor in Children Aged ≥6 Years with Cystic Fibrosis Heterozygous for F508del and a Minimal Function Mutation: Results from a 96-Week Open-Label Extension Study. Eur. Respir. J..

[12] Basile M., Polo J., Henthorne K., DeCelie-Germana J., Galvin S., Wang J. (2024). The Impact of Elexacaftor/Tezacaftor/Ivacaftor on Cystic Fibrosis Health-Related Quality of Life and Decision-Making about Daily Treatment Regimens: A Mixed Methods Exploratory Study. Ther. Adv. Chronic Dis..

[13] Fajac I., Daines C., Durieu I., Goralski J.L., Heijerman H., Knoop C., Majoor C., Bruinsma B.G., Moskowitz S., Prieto-Centurion V. (2023). Non-Respiratory Health-Related Quality of Life in People with Cystic Fibrosis Receiving Elexacaftor/Tezacaftor/Ivacaftor. J. Cyst. Fibros..

[14] Maritescu A., Pescaru C.C., Crisan A.F., Stoicescu E.R., Oancea C., Iacob D. (2025). The Effects of Progressive Muscle Relaxation on Mental Health and Sleep Quality in Adults with Cystic Fibrosis: A Randomized Controlled Trial. J. Clin. Med..

[15] Piehler L., Thalemann R., Lehmann C., Thee S., Röhmel J., Syunyaeva Z., Stahl M., Mall M.A., Graeber S.Y. (2023). Effects of Elexacaftor/Tezacaftor/Ivacaftor Therapy on Mental Health of Patients with Cystic Fibrosis. Front. Pharmacol..

[16] Drai C., van der Woude H.J., Lexmond A.J., van den Hoorn T., Mol P.G.M., Leacy F.P., Przybyszewska A., Sepodes B., Castelnovo T. (2026). The European Medicines Agency’s Review of Elexacaftor/Tezacaftor/Ivacaftor: Extending Its Use to All People with Cystic Fibrosis Aged 2 Years and Older Who Do Not Have Two Class I CFTR Variants. Eur. Respir. J..

[17] Ministry of Health, National Health Insurance House (2022). Order No. 1 206/233/2022 on the Amendment and Supplementation of Annexes No. 1 and 2 to Order No. 564/499/2021 for the Approval of Therapeutic Protocols.

[18] Ministry of Health, National Health Insurance House (2022). Order No. 3322/909/2022 on the Amendment and Supplementation of Annexes No. 1 and 2 to Order No. 564/499/2021 for the Approval of Therapeutic Protocols.

[19] Ministry of Health, National Health Insurance House (2025). Order No. 1636/1849/2025 on the Amendment and Supplementation of Annexes No. 1 and 2 to Order No. 564/499/2021 for the Approval of Therapeutic Protocols.

[20] Ministry of Health, National Health Insurance House (2022). Order No. 702/133/2022 on the Amendment and Supplementation of Annex No. 1 to Order No. 564/499/2021 for the Approval of Therapeutic Protocols.

[21] Bodnar R., Kadar L., Holics K., Ujhelyi R., Kovacs L., Bolbas K., Szekely G., Gyurkovits K., Solyom E., Meszaros A. (2014). Factors Influencing Quality of Life and Disease Severity in Hungarian Children and Young Adults with Cystic Fibrosis. Ital. J. Pediatr..

[22] Borawska-Kowalczyk U., Bodnar R., Meszaros A., Sands D. (2015). Comparison of Health-Related Quality of Life among Children with Cystic Fibrosis and Their Parents in Two Eastern European Countries. J. Cyst. Fibros. Off. J. Eur. Cyst. Fibros. Soc..

[23] Walicka-Serzysko K., Postek M., Mielus M., Borawska-Kowalczyk U., Milczewska J., Zybert K., Wozniacki Ł., Wołkowicz A., Sands D. (2025). Comprehensive Evaluation of Elexacaftor/Tezacaftor/Ivacaftor in Paediatric Cystic Fibrosis: Nutritional, Pulmonary, and Quality-of-Life Outcomes. J. Clin. Med..

[24] Humaj-Grysztar M., Rachel M., Bonior J. (2024). Polish Cystic Fibrosis Patients’ Health-Related Quality of Life and Its Influencing Factors: A Cross-Sectional, Single-Centre Study. Healthcare.

[25] Anton-Paduraru D.-T., Moscalu M., Oltean C., Trandafir L.M.S., Ciubara A. (2015). 283 Psycho-Social Aspects in Children with Cystic Fibrosis from a Regional Centre in North-Eastern Romania. J. Cyst. Fibros..

[26] Dediu M., Ciuca I.M., Marc M.S., Boeriu E., Pop L.L. (2021). Factors Influencing Lung Function in Patients with Cystic Fibrosis in Western Romania. J. Multidiscip. Healthc..

[27] Stemler S. (2000). An overview of content analysis. Pract. Assess. Res. Eval..

[28] Nichols D.P., Paynter A.C., Heltshe S.L., Donaldson S.H., Frederick C.A., Freedman S.D., Gelfond D., Hoffman L.R., Kelly A., Narkewicz M.R. (2022). Clinical Effectiveness of Elexacaftor/Tezacaftor/Ivacaftor in People with Cystic Fibrosis: A Clinical Trial. Am. J. Respir. Crit. Care Med..

[29] Ibrahim H., O’Mahony A.T., Deasy K.F., Waldron M., McCarthy M., Dorgan J., Fleming C., Howlett C., O’Riordan E., McCarthy Y. (2025). Real-World Improvement in Ultra-Low-Dose Thoracic Computed Tomography Scores, Systemic Inflammatory Markers and Patient-Reported Outcome Measures after Elexacaftor/Tezacaftor/Ivacaftor Treatment. ERJ Open Res..

[30] Muilwijk D., van Paridon T.J., van der Heijden D.C., Faber-Bisschop B.M., Ommen D.D.Z., Heijerman H.G.M., van der Ent C.K. (2023). Development and Validation of a Novel Personalized Electronic Patient-Reported Outcome Measure to Assess Quality of Life (Q-LIFE): A Prospective Observational Study in People with Cystic Fibrosis. eClinicalMedicine.

[31] Perrotta N., Fiorito L.A., Gentile R., Vescovo R., Piciocchi A., Troiani P., Poscia R., Cimino G. (2025). Real-World Evaluation of Outcomes and Safety of Elexacaftor/Tezacaftor/Ivacaftor in Pediatric Patients with Cystic Fibrosis: A Retrospective Study. Clin. Transl. Sci..

[32] Kümmerli S., Elviro C.F., Regamey N., Mornand A., Faouzi M., Rochat I., Blanchon S. (2026). Impact of Elexacaftor-Tezacaftor-Ivacaftor on Quality of Life in Children with Cystic Fibrosis. Pediatr. Pulmonol..

[33] Ancel J., Launois C., Perotin J.-M., Ravoninjatovo B., Mulette P., Hagenburg J., Malet J., Griffon M., Carré S., Lebargy F. (2022). Health-Related Quality of Life in Adults with Cystic Fibrosis: Familial, Occupational, Social, and Mental Health Predictors. Healthcare.

[34] Van Citters A.D., Aliaj E., Alvarez J.A., Brown C.D., Cary J., Cravens R., Frederick C.A., Georgiopoulos A.M., Goss C.H., Kazmerski T.M. (2025). Wellness in the Modulator Era: An Observational Study of the Impact of CFTR Modulator Therapy on the Well-Being of People with Cystic Fibrosis. J. Cyst. Fibros. Off. J. Eur. Cyst. Fibros. Soc..

[35] Sagel S.D., Poore T.S., Wagner B.D., Xie J., Heltshe S.L., Cross M., Bratcher P.E., Taylor-Cousar J.L., Wilson A., McBennett K. (2026). Long-term reductions in inflammation in people with cystic fibrosis treated with elexacaftor/tezacaftor/ivacaftor. Ann. Am. Thorac. Soc..

[36] Örlős Z., Barta I., Páska C., Halász A., Antus B. (2025). Impact of Long-Term Elexacaftor/Tezacaftor/Ivacaftor Therapy on Oxidative Stress in Cystic Fibrosis: Early Insights. J. Cyst. Fibros. Off. J. Eur. Cyst. Fibros. Soc..

[37] Lee T.W.R., Duff A.J.A. (2024). Questions and Answers? Depression Symptoms Associated with Elexacaftor/Tezacaftor/Ivacaftor Treatment for Cystic Fibrosis. Am. J. Respir. Crit. Care Med..

[38] Ramsey B., Correll C.U., DeMaso D.R., McKone E., Tullis E., Taylor-Cousar J.L., Chu C., Volkova N., Ahluwalia N., Waltz D. (2024). Elexacaftor/Tezacaftor/Ivacaftor Treatment and Depression-Related Events. Am. J. Respir. Crit. Care Med..

[39] Shoff S.M., Tluczek A., Laxova A., Farrell P.M., Lai H.J. (2013). Nutritional Status Is Associated with Health-Related Quality of Life in Children with Cystic Fibrosis Aged 9–19 Years. J. Cyst. Fibros. Off. J. Eur. Cyst. Fibros. Soc..

[40] Greaney C., Cichy N., O’Sullivan G., Sills D., Cahalan R., Tierney A. (2026). Evolution of Body Image across Treatment Eras: A Systematic Review in Young People and Adults Living with Cystic Fibrosis. Eur. Respir. Rev..

[41] Uchmanowicz I., Jankowska-Polańska B., Rosińczuk J., Wleklik M. (2015). Health-Related Quality of Life of Patients Suffering from Cystic Fibrosis. Adv. Clin. Exp. Med. Off. Organ Wroc. Med. Univ..

[42] Iovănescu G., Pop L.L., Vintilă D.R., Marin K.C., Mogoantă C.A., Lăzureanu D.C., Borugă V.M., Ciucă I.M. (2023). Nasal Polyposis from Cystic Fibrosis in Children—The Experience of a Single Center. Rom. J. Morphol. Embryol..

[43] Marinău C., Csep A., Sava C., Iuhas A., Niulaș L., Szilagyi A., Ritli L., Balmoș A., Jurca C. (2023). Difficulties in the Management of an Askin Tumor in a Pediatric Patient with Cystic Fibrosis: Case Report and Literature Review. Front. Pediatr..

[44] Stan I.V., Miron V.D., Vangheli I.A., Gheorghiu R.M., Streinu-Cercel A., Săndulescu O., Craiu M. (2022). First Case of COVID-19 Treated with Monoclonal Anti-Spike Antibodies in a Patient with Cystic Fibrosis in Romania. Diagnostics.

