# Supplementary material for: Patient-Reported Health-Related Quality of Life in Romanian Patients with Cystic Fibrosis in the Era of Highly Effective CFTR Modulator Therapy: A National Cross-Sectional Mixed-Methods Survey
Source: J Clin Med. 2026 Aug 16;15(16):6328. doi: 10.3390/jcm15166328 (PMC13513152; doi:10.3390/jcm15166328)
Supplement: Supplementary file 1 [file jcm-15-06328-s001.zip › jcm-4453679-supplementary.pdf]

Supplemental Tables

Supplementary Table S1. Categories, codes, frequencies, and illustrative quotations from qualitative content analysis of open-ended responses by CFTR modulator therapy status

Main category	Code / subcategory	n/N (%)	Illustrative quotations
**Currently treated with CFTR modulators**			
1.Overall perceived effect of CFTR modulators	1A Clear improvement	13/49 (26.5%)	"On all levels, there was a very good evolution, without adverse reactions."
	1B No major change	5/49 (10.2%)	
	1C Mixed or uncertain effect	1/49 (2.0%)	
2.Respiratory improvement or burden	2A Less cough / easier breathing	16/49 (32.7%)	"I used to cough often and now with the treatment I don't cough anymore. Pneumonia is rarer."
	2B Fewer respiratory infections / milder viral episodes	14/49 (28.6%)	
	2C Lung function / clinical test improvement	2/49 (4.1%)	
3.Hospitalization and treatment burden	3A Fewer hospitalizations / antibiotics	14/49 (28.6%)	"Hospitalizations have decreased, and overall well-being is much better."
	3B Daily treatment burden remains	4/49 (8.2%)	
	3C Desire to reduce treatment burden	2/49 (4.1%)	
4.Nutrition, growth, gastrointestinal symptoms and other non-GI symptomes	4A Weight gain / better growth	13/49 (26.5%)	"Before starting treatment, she had issues with weight and coughing. After beginning treatment, she gained weight, grew taller, and has not required any further hospitalizations."
	4B Digestive symptoms improved or present	5/49 (10.2%)	
	4C Liver, sweat/sweat test, laboratory improvements	7/49 (14.3%)	
5.Energy, activity, and daily life participation	5A More energy / less fatigue	12/49 (24.5%)	"Before treatment, she would get tired while walking, had a very bad cough, and was very thin."
	5B Better physical activity / school / normal life	6/49 (12.2%)	
	5C Social participation and identity	3/49 (6.1%)	
6.Emotional, mental, and family burden	6A Anxiety / stress / agitation	4/49 (8.2%)	"Life with cystic fibrosis is hard for a teenager to cope with. I am often fed up with swallowing pills and sometimes I don't want to take them anymore. It is difficult to constantly answer questions from my peers: "Why are you so thin if you eat so much?"; "Why can't you come with us?"; "Are you tired, again?"."
	6B Treatment fatigue or frustration	4/49 (8.2%)	
	6C Family/caregiver burden	2/49 (4.1%)	
	6D Stigma or peer misunderstanding	1/49 (2.0%)	
7.Safety, side effects, and comorbidities	7A No adverse effects reported	1/49 (2.0%)	"After starting the modulator, the respiratory problems disappeared

	7B Side effects / new symptoms	1/49 (2.0%)	completely. The side effects included stomach pain, occasional digestive issues, skin rashes, and fatigue."
	7C Other comorbidities	2/49 (4.1%)	
8.Access, future hopes, and care experience	8A Positive care-team experience	1/49 (2.0%)	"Throughout our cystic fibrosis journey, we have been fortunate to have the best professors by our side, together with an entire team that is wholeheartedly committed to the fight against cystic fibrosis."
**Not currently receiving modulator therapy**			
8.Access, future hopes, and care experience	8B Waiting for modulators / genetic eligibility	2/3 (66.7%)	"I hope for new medicines and better health."
	8C Desire for new medicines / better access	1/3 (33.3%)	

Values are presented as n/N (%), where N represents the number of respondents with analyzable open-ended qualitative content in each subgroup. For respondents currently receiving CFTR modulator therapy, N = 49; for respondents not currently receiving CFTR modulator therapy, N = 3. Codes were non-mutually exclusive, meaning that a single response could be assigned to more than one code; therefore, percentages do not sum to 100%. The not-currently-treated subgroup included respondents who had never received CFTR modulator therapy or had interrupted/stopped treatment. Illustrative quotations were selected to represent the content of each main category and were translated into English. The table follows qualitative content analysis principles, using categories and subcategories to describe the meaning of responses and selected quotations to support the link between data and interpretation.

Supplementalry Table S2. Exploratory associations across non-respiratory CFQ-R domains

CFQ-R domain	BMI z-score, age 6–13 years	BMI, age ≥ 14 years	CFTR therapy status: currently treated; *not currently treated	F508del status: at least one F508del** variant; no F508del	ppFEV1 >70% vs 40–69% predicted	IV antibiotic-treated pulmonary exacerbation in previous 12 months
Physical functioning	n=40, ρ^=0.14, p=0.403	n=17, ρ=0.48, **p=0.050**	n=52/9, p=0.051	n=50/8, p**=0.010**	n=31/6, **p=0.014**	n=39/20, **p<0.001**
Emotional functioning	n=40, ρ=-0.12, p=0.441	n=17, ρ=0.66, **p=0.004**	n=52/9, p=0.208	n=50/8, p=0.125	n=31/6, p=0.059	n=39/20, **p=0.011**
Vitality	n=38, ρ=-0.12, p=0.455	n=17, ρ=0.37, p=0.149	n=49/8, **p=0.014**	n=47/8, p=0.078	n=29/6, p=0.077	n=37/19, **p=0.004**
School functioning†	n=38, ρ=0.12, p=0.466	—	n=33/6, p=0.421	n=32/7, **p=0.044**	n=19/3, p=0.176	n=24/14, p=0.603
Role functioning††	—	n=17, ρ=0.64, **p=0.006**	n=16/2, **p=0.024**	—	n=10/3, p=0.433	n=13/5, **p=0.015**
Eating disturbances	n=40, ρ=0.14, p=0.377	n=17, ρ=0.21, p=0.422	n=52/9, p=0.237	n=50/8, **p=0.002**	n=31/6, p=0.051	n=39/20, p=0.106
Treatment burden	n=40, ρ=-0.28, p=0.082	n=17, ρ=0.46, p=0.066	n=52/9, p=0.750	n=50/8, p=0.494	n=31/6, p=0.073	n=39/20, p=0.193
Health perceptions	n=38, ρ=-0.12, p=0.479	n=17, ρ=0.45, p=0.072	n=49/8, p=**0.034**	n=47/8, **p=0.004**	n=29/6, **p=0.019**	n=37/19, **p=0.001**
Social functioning†††	—	n=17, ρ=0.47, p=0.057	n=19/3, p=0.362	—	n=12/3, p=0.247	n=15/6, p=0.391
Body image	n=40, ρ=0.07, p=0.688	n=17, ρ=0.57, **p=0.016**	n=52/9, p=0.298	n=50/8, **p=0.013**	n=31/6, **p=0.025**	n=39/20, p=0.125
Weight	n=38, ρ=-0.02, p=0.921	n=17, ρ=0.69, **p=0.002**	n=49/8, p=0.107	n=47/8, p=0.429	n=29/6, p=0.545	n=37/19, p=0.398
Digestive symptoms	n=40, ρ=0.03, p=0.847	n=17, ρ=0.42, p=0.089	n=52/9, p=0.458	n=50/8, p=0.410	n=31/6, p=0.933	n=39/20, p=0.564

Abbreviations: BMI, body mass index; CFTR, cystic fibrosis transmembrane conductance regulator; CFQ-R, Cystic Fibrosis Questionnaire-Revised; ppFEV1, percent predicted forced expiratory volume in 1 second.

For participants aged 6–13 years, analyses combined parent-proxy responses and self-reports from children aged 12–13 years when the corresponding CFQ-R domain was available in both questionnaire versions; domains available in only one questionnaire version were analyzed only for eligible respondents.

Exploratory analyses were unadjusted and based on available cases. For BMI and BMI z-scores results are presented as Spearman's rank correlation coefficient **(ρ)** with corresponding p-values. For CFTR therapy status, F508del status, ppFEV1 category and IV antibiotic-treated pulmonary exacerbations in the previous 12 months, p-values were calculated using the Mann–Whitney U test. Respondents with unknown ppFEV1 category or missing data for a given variable were excluded from the corresponding analysis.
Bold value indicates statistical significance at $p < 0.05$.

^Spearman's rho.

* The "not currently treated" CFTR modulator group included one participant who had interrupted treatment at the time of questionnaire completion.

** For F508del status, homozygous and heterozygous F508del participants were grouped as having at least one F508del variant; participants with unknown F508del status were excluded from this comparison.

†School functioning is available only in the parent-reported age 6-13 years questionnaire.

††Role functioning is available only for ≥ 14 years, adolescents and adult questionnaire.

†††Social functioning is available in both 12-13 self-report, and the ≥ 14 years, adolescents and adult questionnaire.
